# Primary Peritoneal Pregnancy Requiring Conversion From Laparoscopic to Open Surgery Due to Massive Hemorrhage: A Case Report

**DOI:** 10.7759/cureus.103447

**Published:** 2026-02-11

**Authors:** Rieko Okubo, Tomomi Egawa-Takata, Mayako Goto, Kensuke Hori, Kimihiko Ito

**Affiliations:** 1 Obstetrics and Gynecology, Kansai Rosai Hospital, Amagasaki, JPN

**Keywords:** ectopic pregnancy, laparoscopic surgery, massive hemorrhage, open conversion, peritoneal pregnancy

## Abstract

Ultrasound and magnetic resonance imaging are useful tools for the preoperative diagnosis of peritoneal pregnancy; however, accurate diagnosis before surgery remains challenging. We report a rare case of primary peritoneal pregnancy in which laparoscopic surgery was initiated under the suspicion of tubal pregnancy, but was converted to open surgery due to massive hemorrhage.

A 40-year-old female (gravida 1, para 1) was referred to our hospital at six weeks and six days of gestation for suspected ectopic pregnancy. Despite a serum human chorionic gonadotropin (hCG) level of 10,060 mIU/mL, no intrauterine gestational sac was identified. Imaging revealed a gestational sac-like hematoma adjacent to the uterus. Laparoscopic surgery was planned for a presumed tubal pregnancy. Intraoperatively, a gestational sac was found in the pouch of Douglas, leading to the diagnosis of peritoneal pregnancy. During the attempted removal of the trophoblastic tissue, massive hemorrhage occurred, necessitating conversion to open surgery. The total blood loss was 2,450 mL, and a blood transfusion was required. The postoperative course was uneventful, and serum hCG levels declined appropriately after surgery.

## Introduction

Ectopic pregnancy is a gynecological emergency that can cause acute abdominal symptoms and life-threatening hemorrhage. With the widespread use of assisted reproductive technologies, the incidence of ectopic pregnancy has increased; however, peritoneal pregnancy remains extremely rare. Abdominal pregnancy is defined as the implantation of a fertilized ovum on the peritoneal surface and accounts for approximately 0.9-1.3% of all ectopic pregnancies [1,2].

The maternal mortality rate associated with peritoneal pregnancy has been reported to be approximately 5.1 per 1,000 pregnancies, which is higher than that of other ectopic pregnancies [3].

Preoperative diagnosis of peritoneal pregnancy using ultrasonography or magnetic resonance imaging is often difficult. Consequently, diagnosis is frequently made intraoperatively, often during laparotomy or laparoscopy. We report a rare case of primary peritoneal pregnancy in which laparoscopic surgery was initiated for suspected tubal pregnancy but required conversion to open surgery because of uncontrollable hemorrhage.

## Case presentation

**Figure 1:**
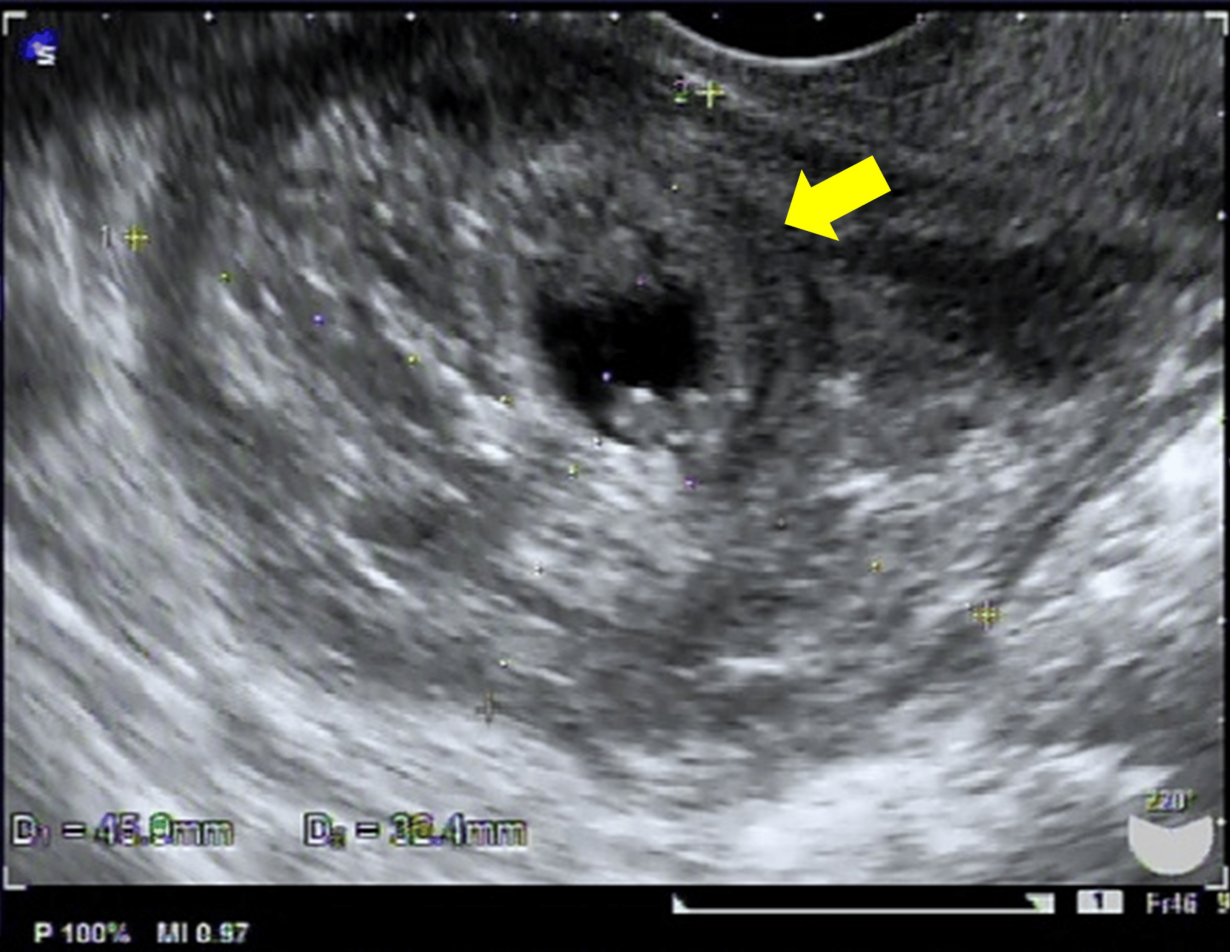
Preoperative transvaginal ultrasound examination. A hematoma-like mass measuring approximately 45 × 32 mm was observed on the right dorsal side of the uterus, containing a gestational sac and embryo (arrow) without detectable fetal cardiac activity.

**Figure 2:**
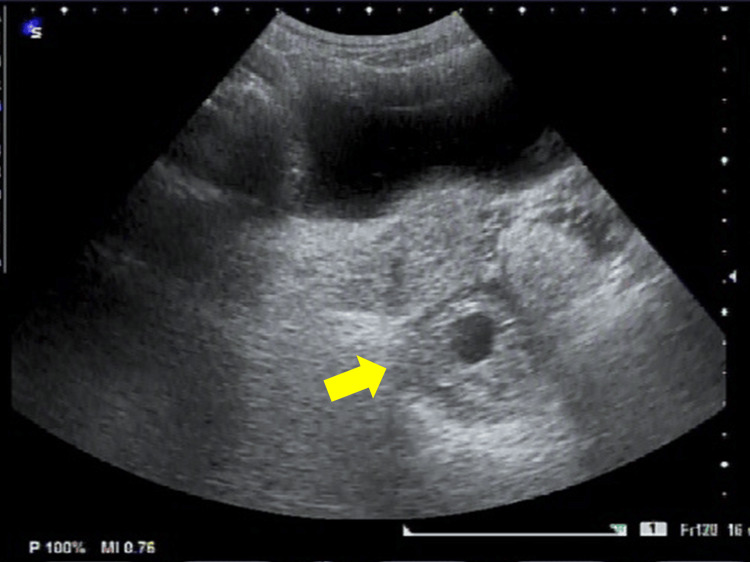
Preoperative transabdominal ultrasound. A gestational sac (arrow) is visible on the right posterior side of the uterus.

A 40-year-old female (gravida 1, para 1) with no significant medical history was referred to our hospital at six weeks and six days of gestation for suspected ectopic pregnancy. At her initial presentation, her serum human chorionic gonadotropin (hCG) level was 10,060 mIU/mL, and no intrauterine gestational sac was detected on transvaginal ultrasonography. A hematoma-like mass measuring approximately 45 × 32 mm was observed on the right dorsal side of the uterus, containing a gestational sac and embryo without detectable fetal cardiac activity (Figures 1, 2).

Based on these findings, right tubal pregnancy was suspected, and laparoscopic surgery was planned.

**Figure 3:**
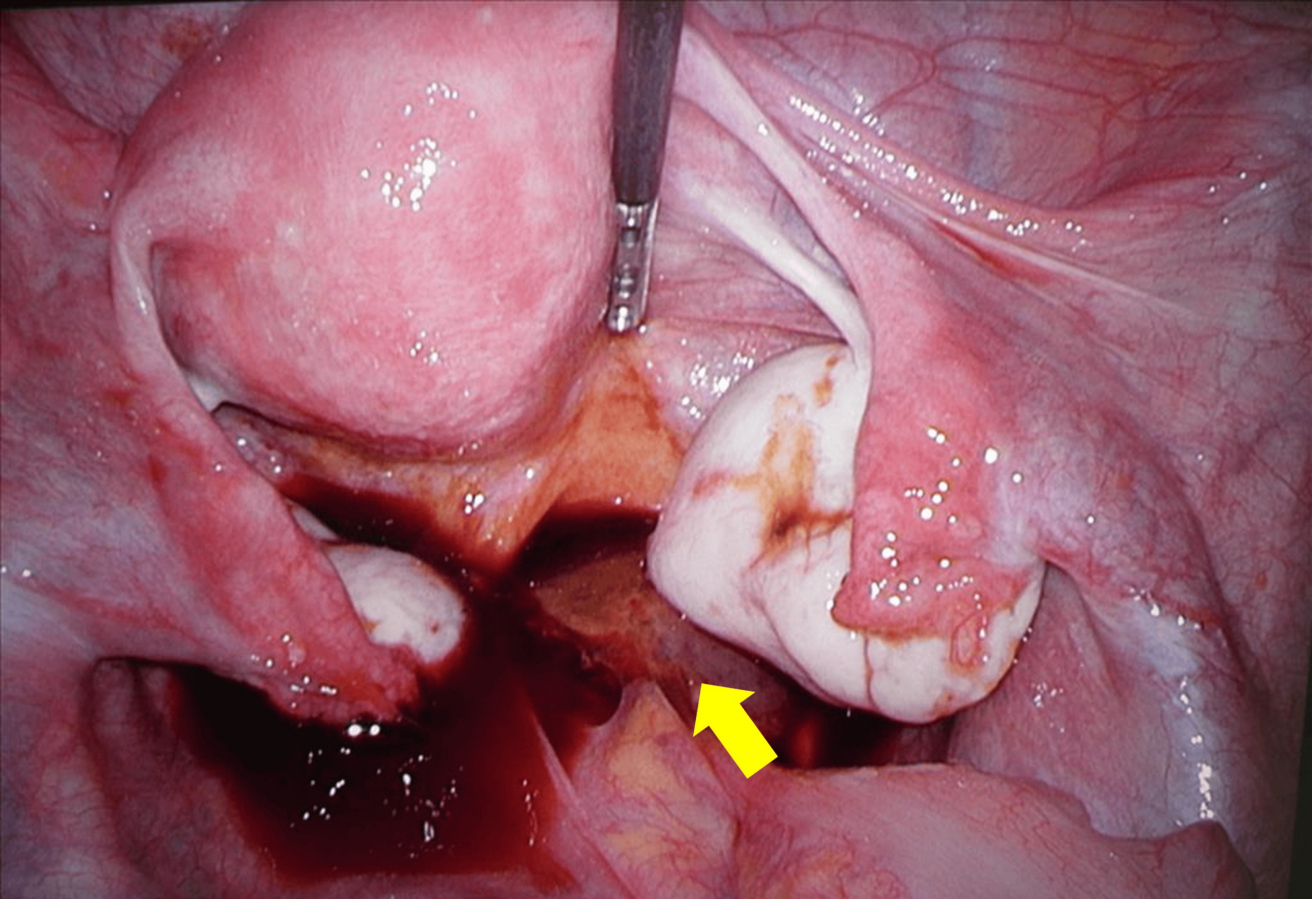
Intra-abdominal findings. A gestational sac (arrow) was identified on the peritoneal surface on the right side of the rectum, corresponding to the pouch of Douglas.

Laparoscopic surgery was performed using a four-port technique. Intra-abdominal inspection revealed a right ovarian cyst; however, the uterus and bilateral adnexa appeared macroscopically normal. A gestational sac was identified on the peritoneal surface on the right side of the rectum, corresponding to the pouch of Douglas, accompanied by a small amount of bleeding (Figure 3). A diagnosis of peritoneal pregnancy was made.

**Figure 4:**
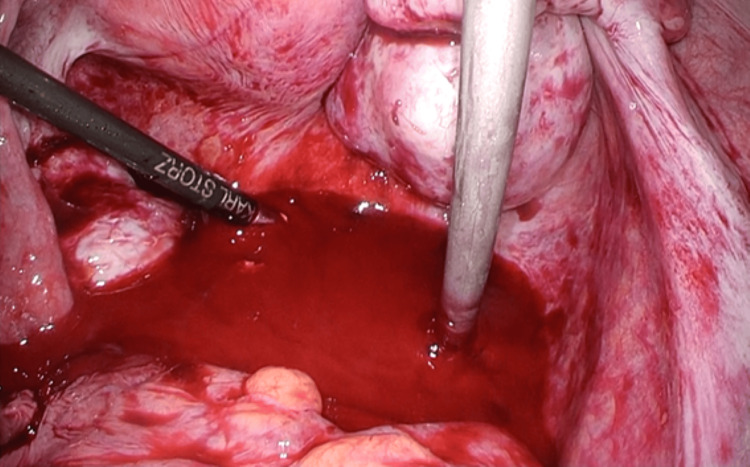
Intraoperative findings. When attempting removal using grasping forceps, the tissue ruptured during separation of the trophoblastic tissue from the retroperitoneal cavity, resulting in massive hemorrhage.

The gestational sac appeared partially detached, and removal was attempted using grasping forceps. During the dissection of the trophoblastic tissue from the retroperitoneal space, the tissue ruptured, resulting in massive hemorrhage (Figure 4). Endoscopic hemostasis using cauterization was attempted but proved unsuccessful. Therefore, the procedure was converted to open surgery 28 minutes after the start of laparoscopy.

A midline incision extending from the umbilicus to the suprapubic region was made. The trophoblastic tissue was manually dissected and removed. Venous bleeding from the pouch of Douglas was observed, and inspection revealed erosion of the retroperitoneal tissue on the right side of the rectum with exposure of venous structures. Hemostasis was achieved by suturing the cavity with absorbable monofilament sutures, followed by application of a fibrinogen-containing hemostatic agent.

The total operative time was two hours and 26 minutes, and the estimated blood loss was 2,450 mL. Six units of packed red blood cells and six units of fresh frozen plasma were transfused intraoperatively and postoperatively. Histopathological examination confirmed the presence of chorionic villi.

A 6.5 mm drain was placed in the Douglas pouch. The drain was removed on the third postoperative day. After confirming normal bowel motility, oral feeding was initiated at noon on the day after surgery. Prophylactic antibiotics were administered according to institutional protocol. No postoperative renal or neurological complications were observed. The postoperative course was uneventful, and the patient was discharged on postoperative day nine. Serum hCG levels decreased promptly and became undetectable one month after surgery (Table 1).

**Table 1:** Blood test findings. WBC: white blood cells; HB: hemoglobin; PLT: platelets; PT: prothrombin time; INR: international normalized ratio; APTT: activated partial thromboplastin time; hCG: human chorionic gonadotropin; CRP: C-reactive protein.

Parameters	Preoperative	Postoperative	Postoperative day1	On discharge	One-month post-op	Normal range
WBC (10^3^/μL)	3.7	3.4	5.2	2.9	4.4	3.3-8.6
HB (g/dl)	13.1	7.5	9.5	9.3	12.7	11.6-14.8
PLT (10^3^/μL)	179	115	110	187	162	158-348
PT (seconds)	13.0	16.6	14.7	-	-	10.9-13.3
INR	1.08	1.39	1.21	-	-	0.8-1.2
APTT (seconds)	33.0	29.5	28.0	-	-	24.7-34.7
Fibrinogen (mg/dl)	-	125	229	-	-	200-400
hCG (mIU/ml)	10060.4	-	2161.6	358.2	1.7	0-2.7
Creatinine (mg/dl)	0.55	-	0.61	0.57	0.69	0.46-0.79
CRP (mg/dl)	<0.1	-	4.3	0.4	<0.1	0-0.14

## Discussion

Peritoneal pregnancy is a rare form of ectopic pregnancy, accounting for approximately 0.9-1.3% of all ectopic pregnancies [1,2]. The maternal mortality rate associated with peritoneal pregnancy has been reported to be approximately 5.1 per 1,000 pregnancies, which is higher than that of other ectopic pregnancies [3].

Peritoneal pregnancy is classified as either primary or secondary. Secondary peritoneal pregnancy occurs when a tubal or ovarian pregnancy is expelled into the peritoneal cavity following abortion or rupture and subsequently reimplants on the peritoneal surface. In contrast, primary peritoneal pregnancy is defined as direct implantation of the fertilized ovum onto the peritoneal surface.

Studdiford proposed three diagnostic criteria for primary peritoneal pregnancy: (1) normal bilateral fallopian tubes and ovaries; (2) absence of a uteroperitoneal fistula; and (3) implantation limited exclusively to the peritoneal surface [4]. Friedrich later added a fourth criterion requiring gestational age to be less than 12 weeks [5]. In the present case, all four criteria were fulfilled, supporting the diagnosis of primary peritoneal pregnancy.

Several risk factors for peritoneal pregnancy have been reported, including intrauterine contraceptive device use, endometriosis, pelvic inflammatory disease, and assisted reproductive technologies [2,6]. In this case, the pregnancy was spontaneous, and none of these risk factors were identified.

Preoperative diagnosis of peritoneal pregnancy remains challenging and is often made during laparotomy or laparoscopy. Ultrasonographic findings suggestive of peritoneal pregnancy include absence of an intrauterine gestational sac, absence of tubal enlargement or complex adnexal mass, a gestational sac surrounded by bowel loops and separated by peritoneum, and marked mobility of the gestational sac upon pressure applied with a transvaginal probe [7]. In the present case, peritoneal pregnancy was not suspected preoperatively, and adnexal pregnancy was presumed. Retrospectively, because the patient’s vital signs were stable, preoperative magnetic resonance imaging might have contributed to earlier diagnosis.

Importantly, this case illustrates that even early primary peritoneal pregnancy may be associated with unexpected massive hemorrhage. Although laparoscopic surgery is often selected for suspected ectopic pregnancy, surgeons should remain aware of the potential limitations of minimally invasive approaches when the implantation site is atypical. This experience may prompt clinicians to reconsider surgical strategy and to be better prepared for open conversion in similar clinical scenarios.

Although a massive transfusion was required, no transfusion-related complications or organ damage were observed. This case underscores the importance of early recognition of uncontrolled bleeding and prompt conversion to laparotomy in peritoneal pregnancy.

## Conclusions

We report a rare case of primary peritoneal pregnancy that was initially misdiagnosed as a tubal pregnancy. Although laparoscopic surgery was initiated, massive hemorrhage necessitated conversion to open surgery. Given the difficulty of preoperative diagnosis and the risk of life-threatening hemorrhage, peritoneal pregnancy should be considered in cases of suspected ectopic pregnancy with atypical imaging findings, and careful surgical planning with readiness for open conversion is essential. This case highlights the importance of anticipating massive hemorrhage in peritoneal pregnancy and emphasizes the need for prompt surgical conversion and multidisciplinary preparedness.
